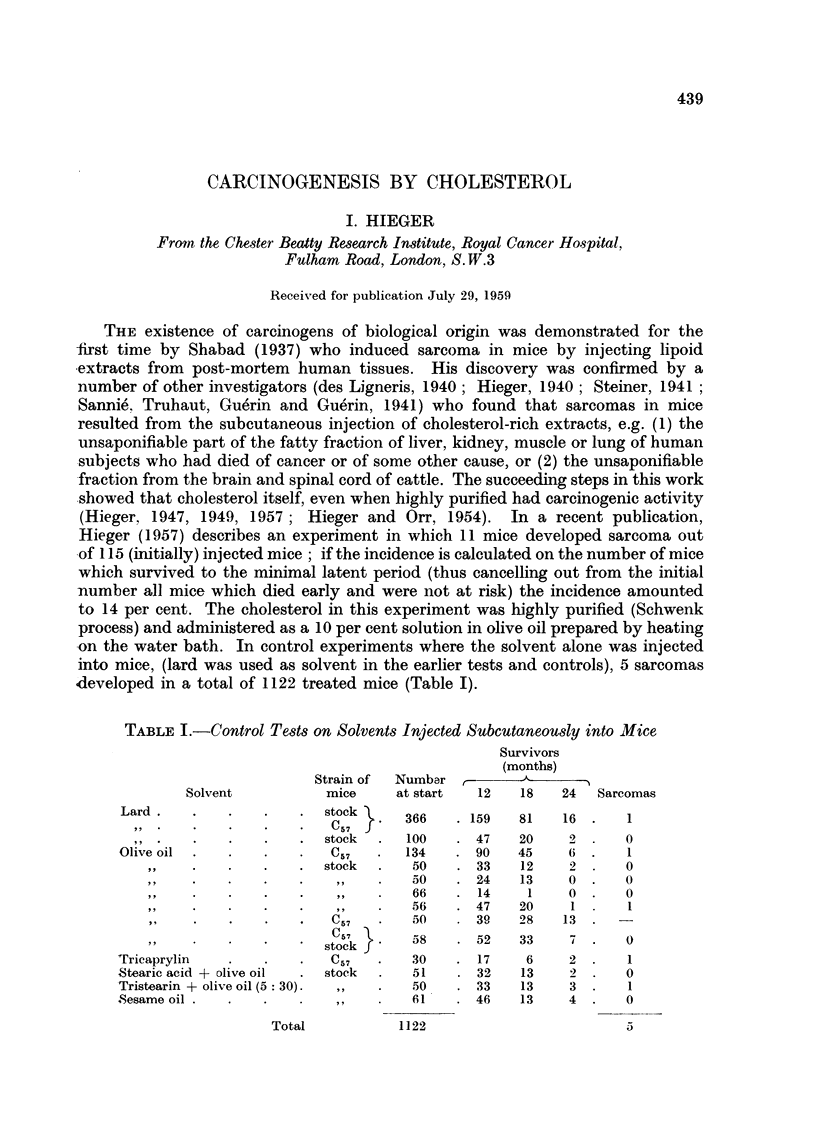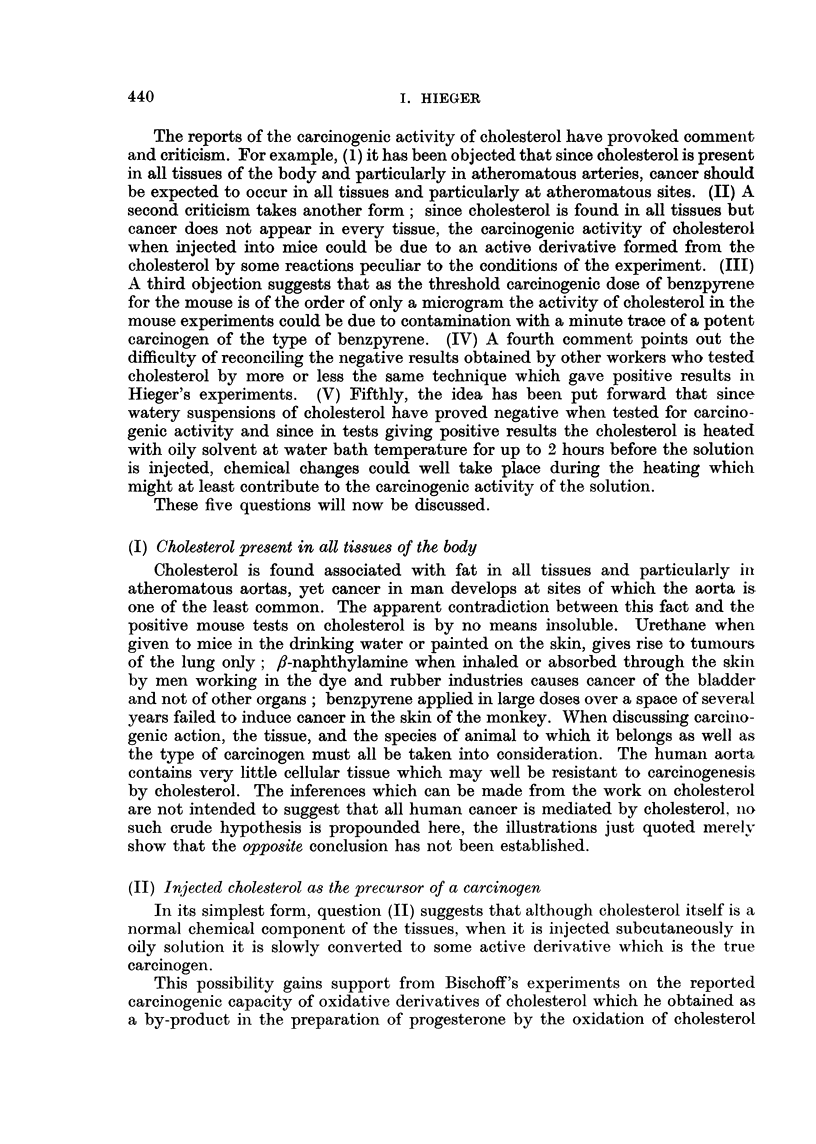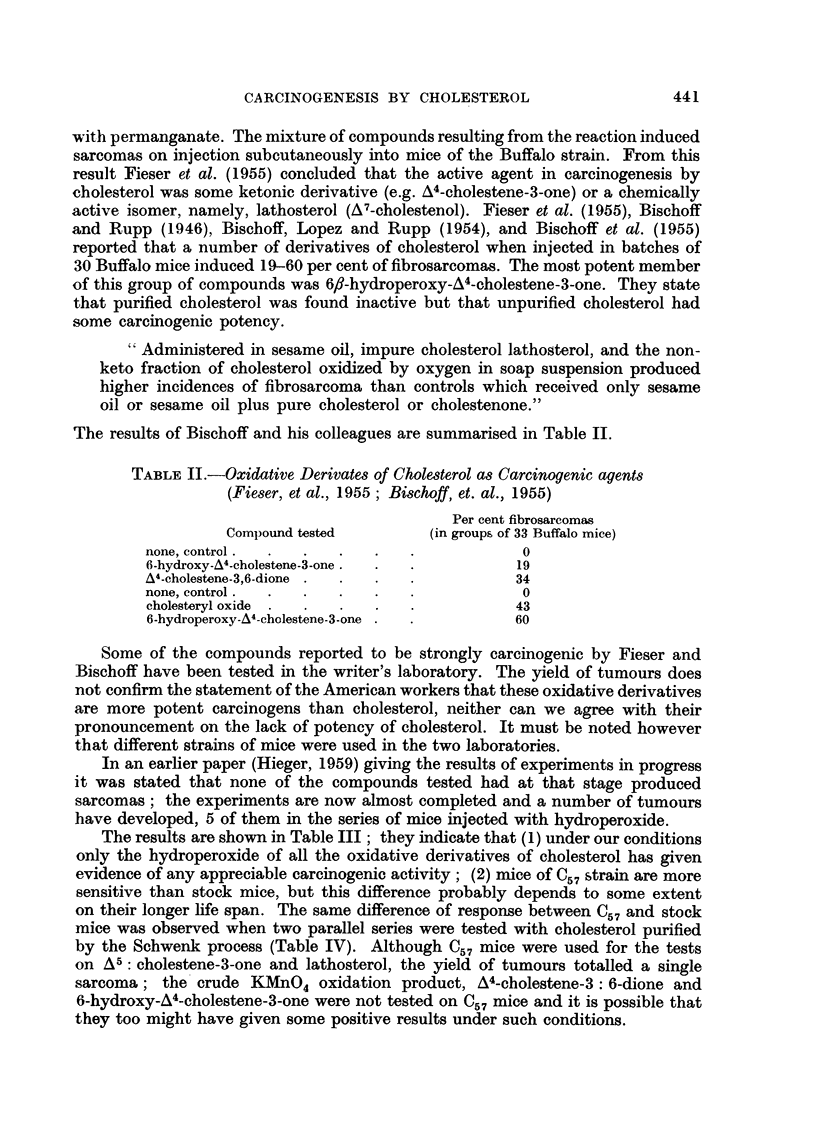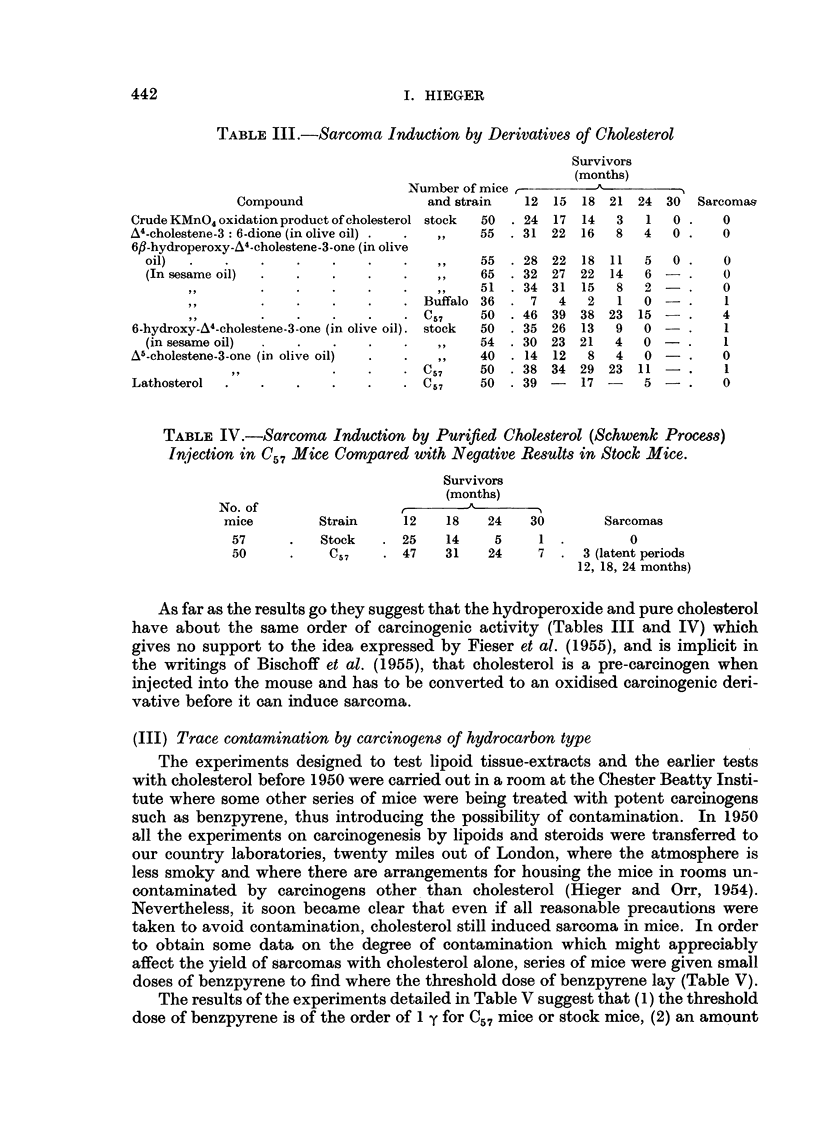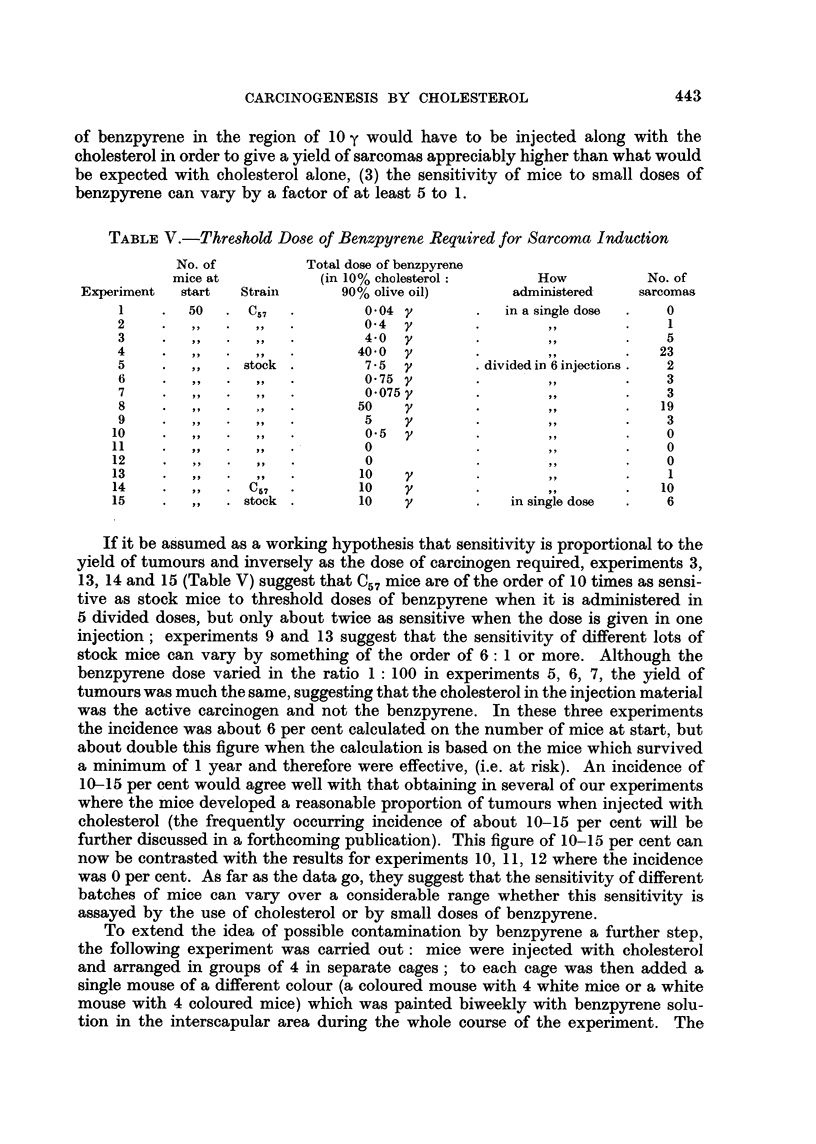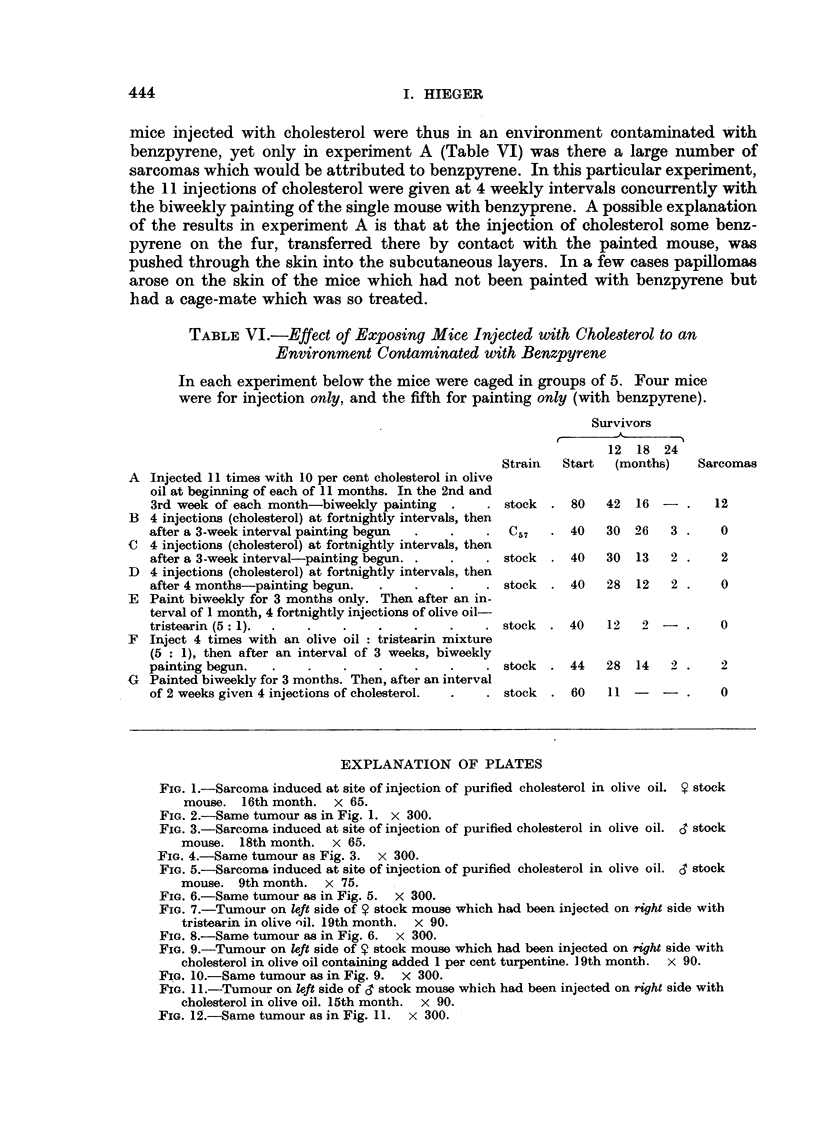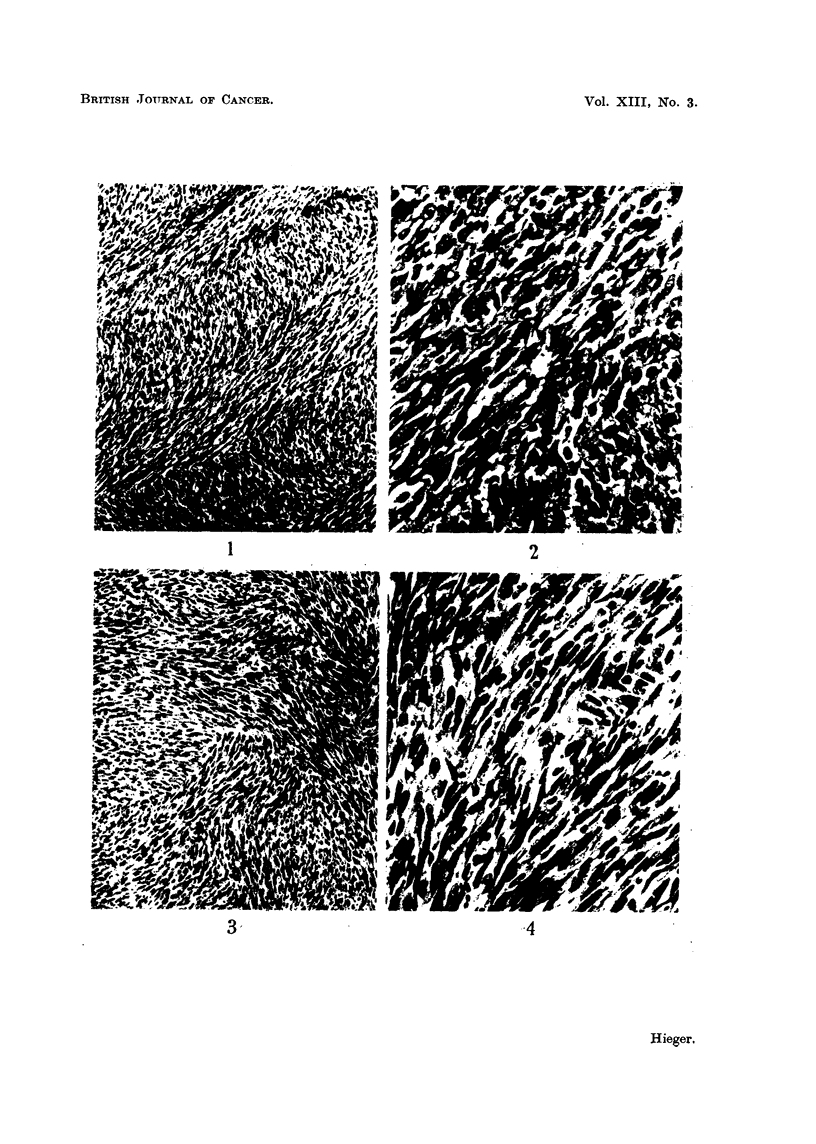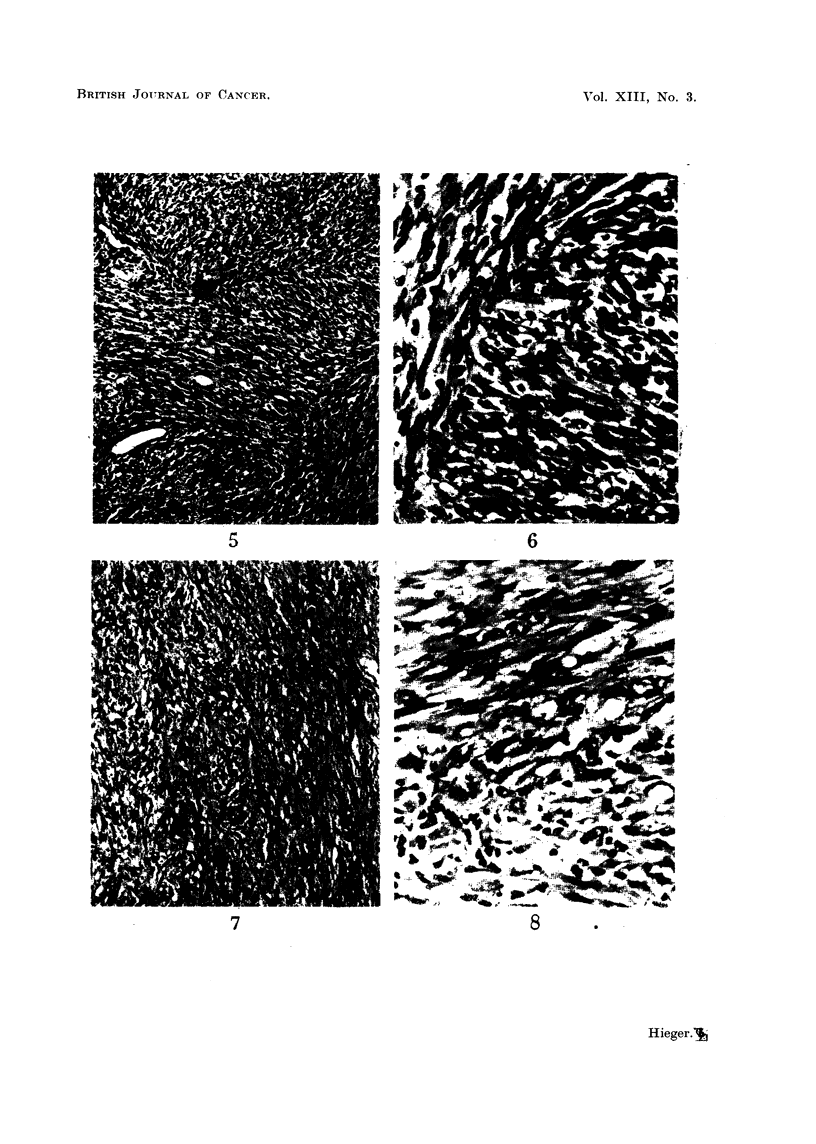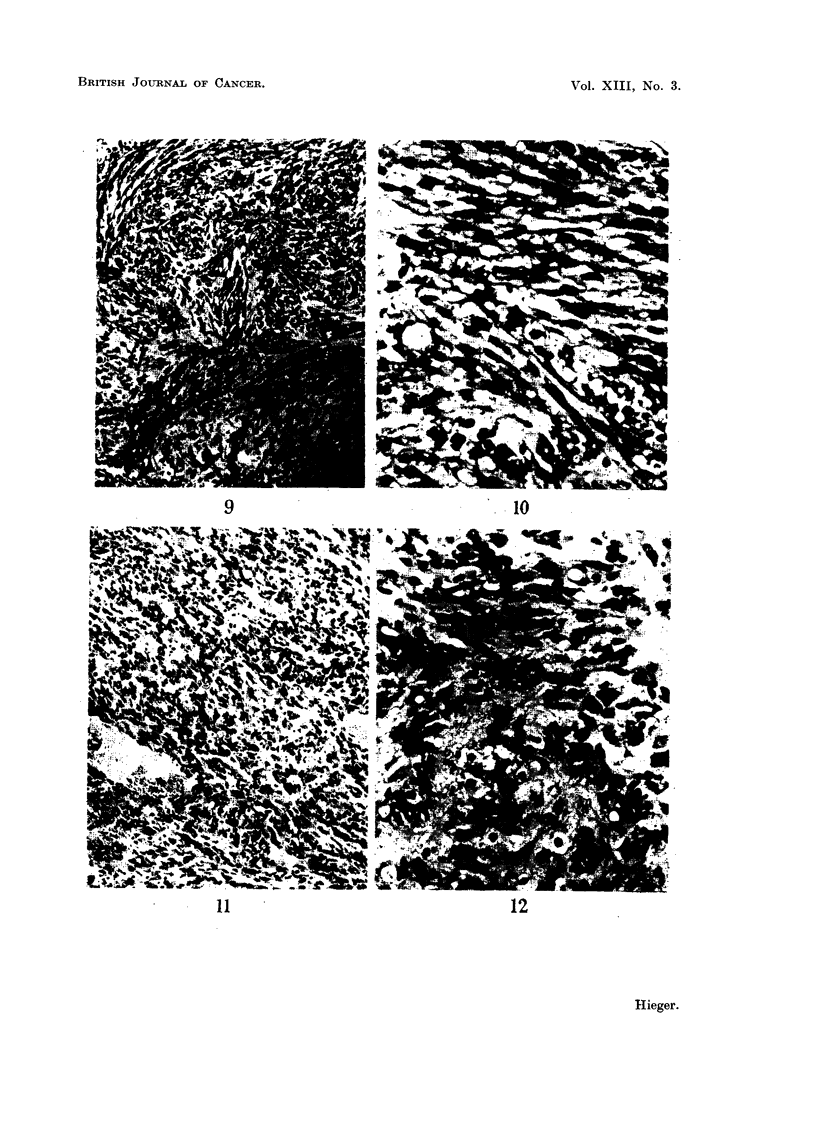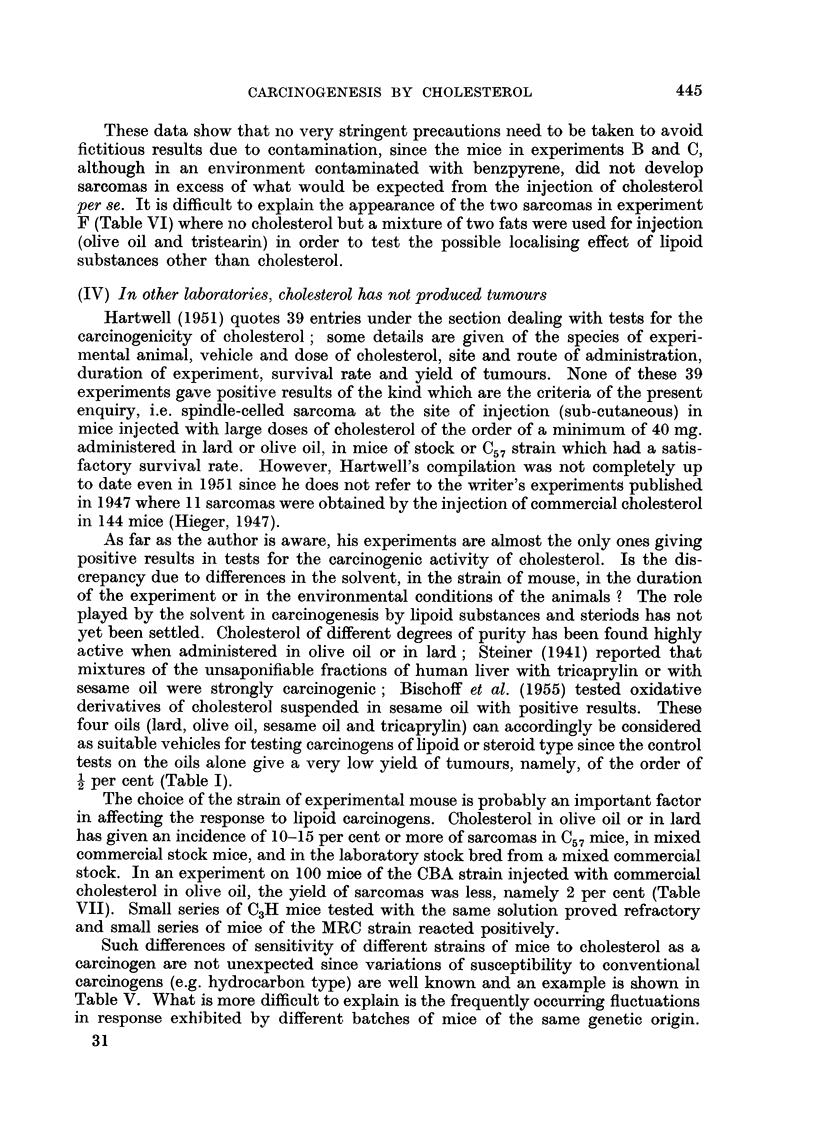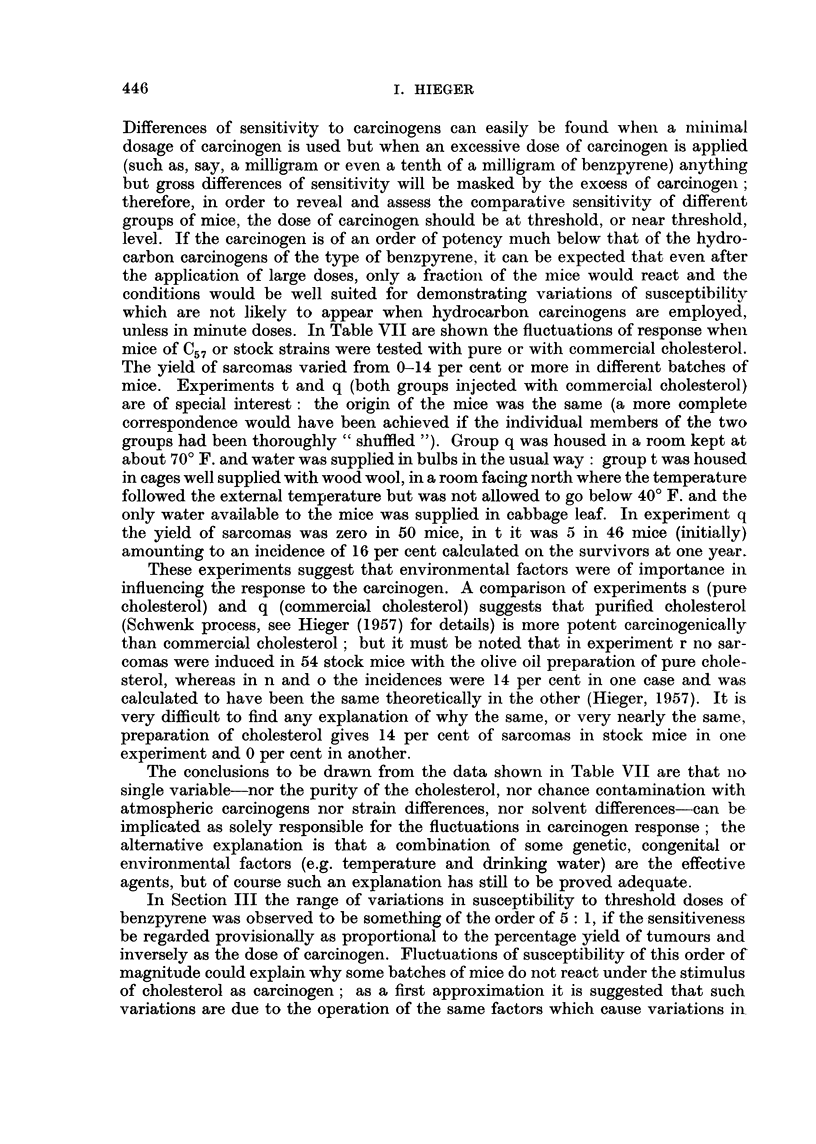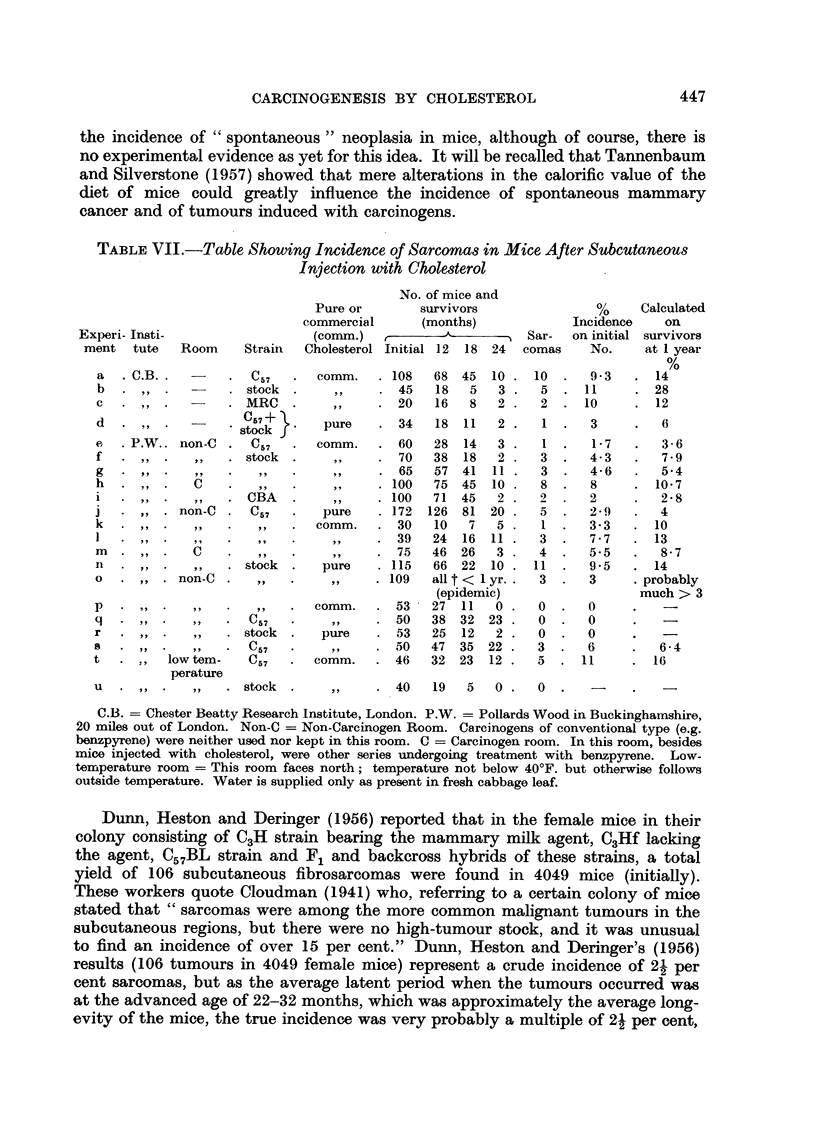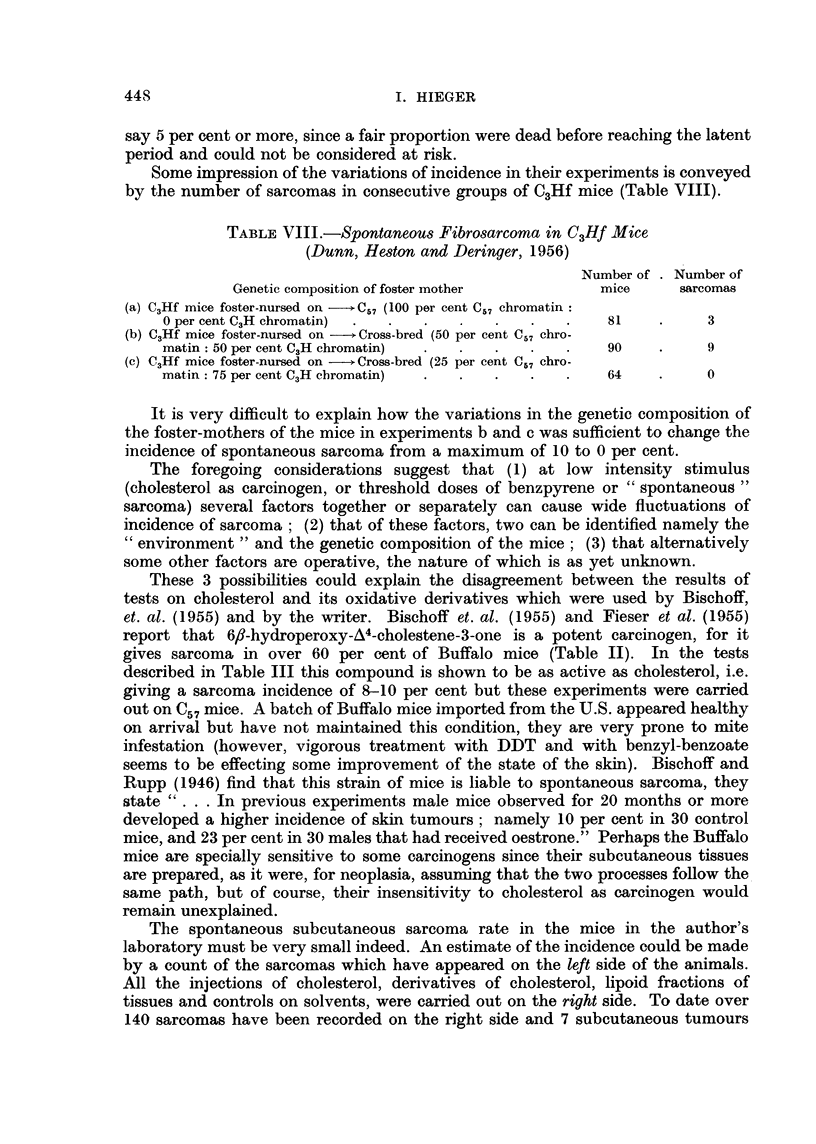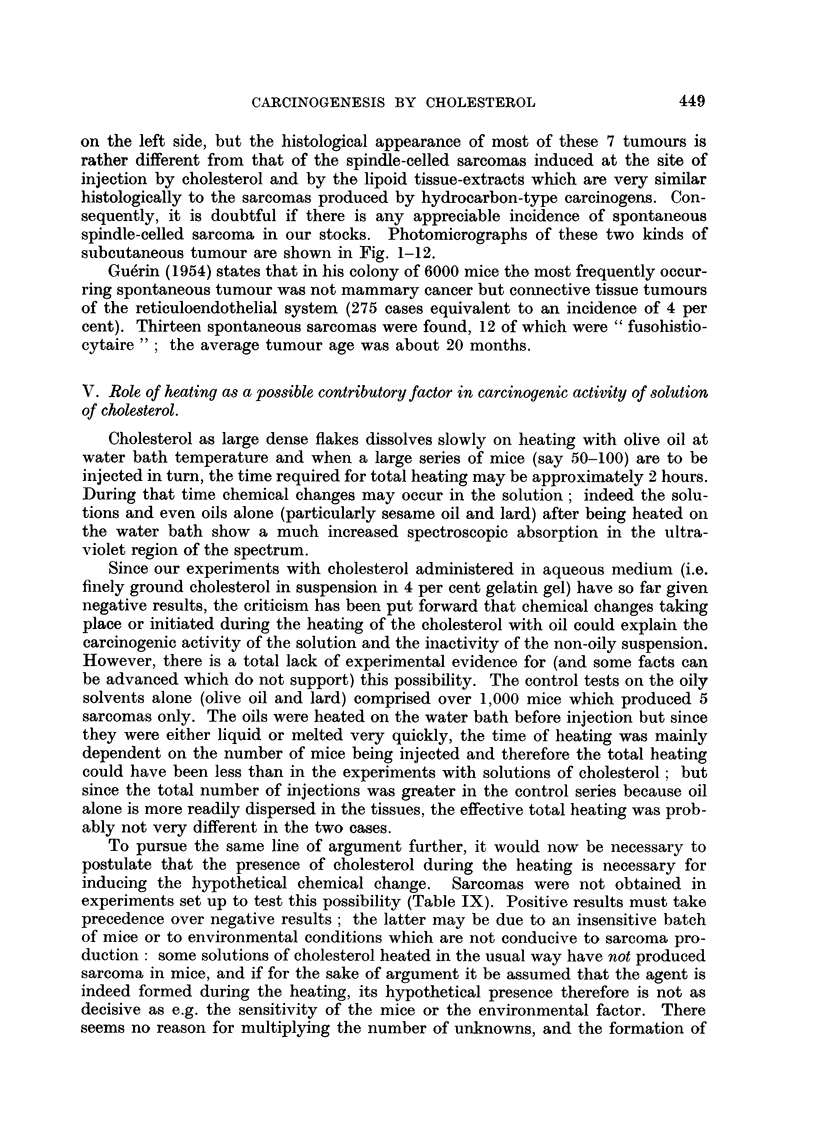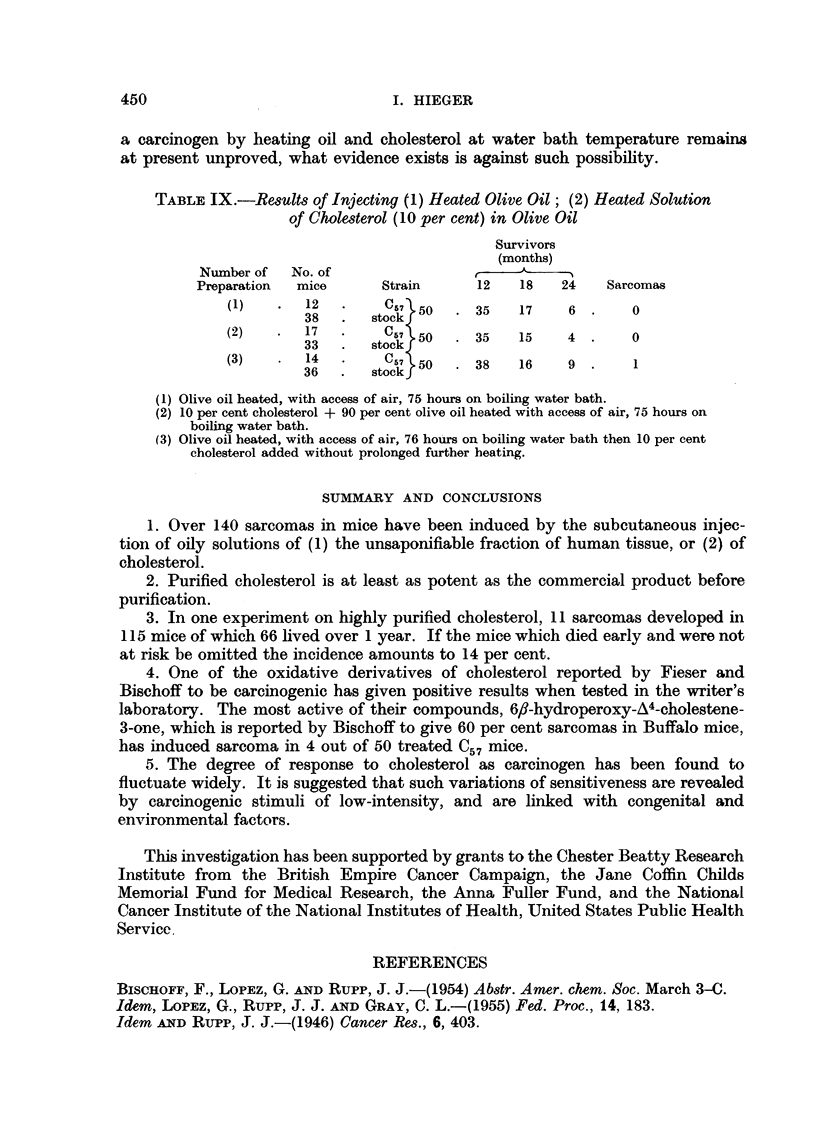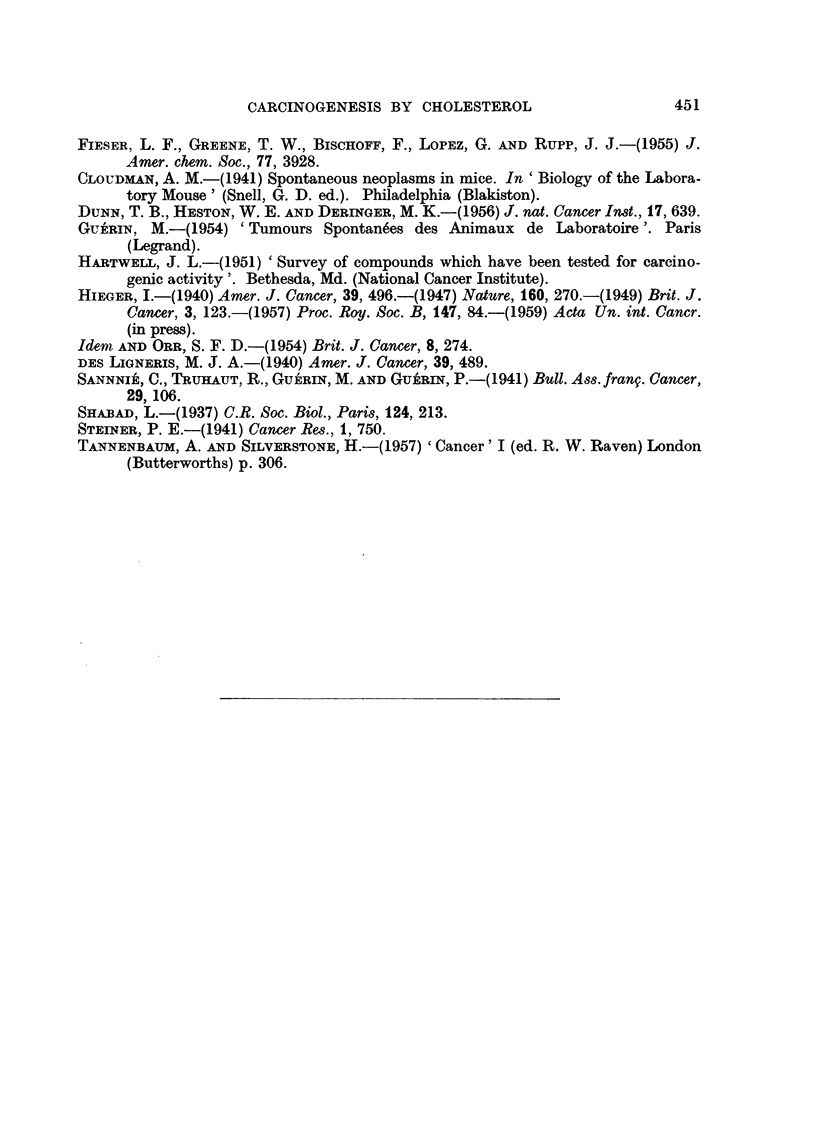# Carcinogenesis by Cholesterol

**DOI:** 10.1038/bjc.1959.49

**Published:** 1959-09

**Authors:** I. Hieger

## Abstract

**Images:**


### CARCINOGENESIS BY CHOLESTEROL

I. HIEGER

*From the Chester Beatty Research Institute, Royal Cancer Hospital, Fulham Road, London, S.W.3*

Received for publication July 29, 1959

THE existence of carcinogens of biological origin was demonstrated for the first time by Shabad (1937) who induced sarcoma in mice by injecting lipoid extracts from post-mortem human tissues. His discovery was confirmed by a number of other investigators (des Ligneris, 1940 ; Hieger, 1940 ; Steiner, 1941 ; Sannié, Truhaut, Guérin and Guérin, 1941) who found that sarcomas in mice resulted from the subcutaneous injection of cholesterol-rich extracts, e.g. (1) the unsaponifiable part of the fatty fraction of liver, kidney, muscle or lung of human subjects who had died of cancer or of some other cause, or (2) the unsaponifiable fraction from the brain and spinal cord of cattle. The succeeding steps in this work showed that cholesterol itself, even when highly purified had carcinogenic activity (Hieger, 1947, 1949, 1957 ; Hieger and Orr, 1954). In a recent publication, Hieger (1957) describes an experiment in which 11 mice developed sarcoma out of 115 (initially) injected mice ; if the incidence is calculated on the number of mice which survived to the minimal latent period (thus cancelling out from the initial number all mice which died early and were not at risk) the incidence amounted to 14 per cent. The cholesterol in this experiment was highly purified (Schwenk process) and administered as a 10 per cent solution in olive oil prepared by heating on the water bath. In control experiments where the solvent alone was injected into mice, (lard was used as solvent in the earlier tests and controls), 5 sarcomas developed in a total of 1122 treated mice (Table I).

TABLE I.—*Control Tests on Solvents Injected Subcutaneously into Mice*

| Solvent | Strain of mice | Number at start | Survivors (months) 12 | 18 | 24 | Sarcomas |
|---|---|---|---|---|---|---|
| Lard<br>,, | stock<br>$C_{57}$ | 366 | 159 | 81 | 16 | 1 |
| ,, | stock | 100 | 47 | 20 | 2 | 0 |
| Olive oil | $C_{57}$ | 134 | 90 | 45 | 6 | 1 |
| ,, | stock | 50 | 33 | 12 | 2 | 0 |
| ,, | ,, | 50 | 24 | 13 | 0 | 0 |
| ,, | ,, | 66 | 14 | 1 | 0 | 0 |
| ,, | ,, | 56 | 47 | 20 | 1 | 1 |
| ,, | $C_{57}$ | 50 | 39 | 28 | 13 | — |
| ,, | $C_{57}$<br>stock | 58 | 52 | 33 | 7 | 0 |
| Tricaprylin | $C_{57}$ | 30 | 17 | 6 | 2 | 1 |
| Stearic acid + olive oil | stock | 51 | 32 | 13 | 2 | 0 |
| Tristearin + olive oil (5 : 30) | ,, | 50 | 33 | 13 | 3 | 1 |
| Sesame oil | ,, | 61 | 46 | 13 | 4 | 0 |
| Total | | 1122 | | | | 5 |

The reports of the carcinogenic activity of cholesterol have provoked comment and criticism. For example, (1) it has been objected that since cholesterol is present in all tissues of the body and particularly in atheromatous arteries, cancer should be expected to occur in all tissues and particularly at atheromatous sites. (II) A second criticism takes another form ; since cholesterol is found in all tissues but cancer does not appear in every tissue, the carcinogenic activity of cholesterol when injected into mice could be due to an active derivative formed from the cholesterol by some reactions peculiar to the conditions of the experiment. (III) A third objection suggests that as the threshold carcinogenic dose of benzpyrene for the mouse is of the order of only a microgram the activity of cholesterol in the mouse experiments could be due to contamination with a minute trace of a potent carcinogen of the type of benzpyrene. (IV) A fourth comment points out the difficulty of reconciling the negative results obtained by other workers who tested cholesterol by more or less the same technique which gave positive results in Hieger's experiments. (V) Fifthly, the idea has been put forward that since watery suspensions of cholesterol have proved negative when tested for carcinogenic activity and since in tests giving positive results the cholesterol is heated with oily solvent at water bath temperature for up to 2 hours before the solution is injected, chemical changes could well take place during the heating which might at least contribute to the carcinogenic activity of the solution.

These five questions will now be discussed.

(I) *Cholesterol present in all tissues of the body*

Cholesterol is found associated with fat in all tissues and particularly in atheromatous aortas, yet cancer in man develops at sites of which the aorta is one of the least common. The apparent contradiction between this fact and the positive mouse tests on cholesterol is by no means insoluble. Urethane when given to mice in the drinking water or painted on the skin, gives rise to tumours of the lung only ; $\beta$-naphthylamine when inhaled or absorbed through the skin by men working in the dye and rubber industries causes cancer of the bladder and not of other organs ; benzpyrene applied in large doses over a space of several years failed to induce cancer in the skin of the monkey. When discussing carcinogenic action, the tissue, and the species of animal to which it belongs as well as the type of carcinogen must all be taken into consideration. The human aorta contains very little cellular tissue which may well be resistant to carcinogenesis by cholesterol. The inferences which can be made from the work on cholesterol are not intended to suggest that all human cancer is mediated by cholesterol, no such crude hypothesis is propounded here, the illustrations just quoted merely show that the *opposite* conclusion has not been established.

(II) *Injected cholesterol as the precursor of a carcinogen*

In its simplest form, question (II) suggests that although cholesterol itself is a normal chemical component of the tissues, when it is injected subcutaneously in oily solution it is slowly converted to some active derivative which is the true carcinogen.

This possibility gains support from Bischoff's experiments on the reported carcinogenic capacity of oxidative derivatives of cholesterol which he obtained as a by-product in the preparation of progesterone by the oxidation of cholesterol with permanganate. The mixture of compounds resulting from the reaction induced sarcomas on injection subcutaneously into mice of the Buffalo strain. From this result Fieser *et al.* (1955) concluded that the active agent in carcinogenesis by cholesterol was some ketonic derivative (e.g. $\Delta^4$-cholestene-3-one) or a chemically active isomer, namely, lathosterol ($\Delta^7$-cholestenol). Fieser *et al.* (1955), Bischoff and Rupp (1946), Bischoff, Lopez and Rupp (1954), and Bischoff *et al.* (1955) reported that a number of derivatives of cholesterol when injected in batches of 30 Buffalo mice induced 19–60 per cent of fibrosarcomas. The most potent member of this group of compounds was 6$\beta$-hydroperoxy-$\Delta^4$-cholestene-3-one. They state that purified cholesterol was found inactive but that unpurified cholesterol had some carcinogenic potency.

"Administered in sesame oil, impure cholesterol lathosterol, and the non-keto fraction of cholesterol oxidized by oxygen in soap suspension produced higher incidences of fibrosarcoma than controls which received only sesame oil or sesame oil plus pure cholesterol or cholestenone."

The results of Bischoff and his colleagues are summarised in Table II.

TABLE II.—*Oxidative Derivates of Cholesterol as Carcinogenic agents (Fieser, et al.,* 1955 ; *Bischoff, et. al.,* 1955)

| Compound tested | Per cent fibrosarcomas (in groups of 33 Buffalo mice) |
|---|---|
| none, control . . . . . . . | 0 |
| 6-hydroxy-$\Delta^4$-cholestene-3-one . . . | 19 |
| $\Delta^4$-cholestene-3,6-dione . . . . | 34 |
| none, control . . . . . . . | 0 |
| cholesteryl oxide . . . . . . | 43 |
| 6-hydroperoxy-$\Delta^4$-cholestene-3-one . . | 60 |

Some of the compounds reported to be strongly carcinogenic by Fieser and Bischoff have been tested in the writer's laboratory. The yield of tumours does not confirm the statement of the American workers that these oxidative derivatives are more potent carcinogens than cholesterol, neither can we agree with their pronouncement on the lack of potency of cholesterol. It must be noted however that different strains of mice were used in the two laboratories.

In an earlier paper (Hieger, 1959) giving the results of experiments in progress it was stated that none of the compounds tested had at that stage produced sarcomas ; the experiments are now almost completed and a number of tumours have developed, 5 of them in the series of mice injected with hydroperoxide.

The results are shown in Table III ; they indicate that (1) under our conditions only the hydroperoxide of all the oxidative derivatives of cholesterol has given evidence of any appreciable carcinogenic activity ; (2) mice of $C_{57}$ strain are more sensitive than stock mice, but this difference probably depends to some extent on their longer life span. The same difference of response between $C_{57}$ and stock mice was observed when two parallel series were tested with cholesterol purified by the Schwenk process (Table IV). Although $C_{57}$ mice were used for the tests on $\Delta^5$ : cholestene-3-one and lathosterol, the yield of tumours totalled a single sarcoma ; the crude $KMnO_4$ oxidation product, $\Delta^4$-cholestene-3 : 6-dione and 6-hydroxy-$\Delta^4$-cholestene-3-one were not tested on $C_{57}$ mice and it is possible that they too might have given some positive results under such conditions.

TABLE III.—*Sarcoma Induction by Derivatives of Cholesterol*

| Compound | Number of mice and strain | | Survivors (months) 12 | 15 | 18 | 21 | 24 | 30 | Sarcomas |
|---|---|---|---|---|---|---|---|---|---|
| Crude $KMnO_4$ oxidation product of cholesterol | stock | 50 | 24 | 17 | 14 | 3 | 1 | 0 | 0 |
| $\Delta^4$-cholestene-3 : 6-dione (in olive oil) | ,, | 55 | 31 | 22 | 16 | 8 | 4 | 0 | 0 |
| 6$\beta$-hydroperoxy-$\Delta^4$-cholestene-3-one (in olive oil) | ,, | 55 | 28 | 22 | 18 | 11 | 5 | 0 | 0 |
| (In sesame oil) | ,, | 65 | 32 | 27 | 22 | 14 | 6 | — | 0 |
| ,, | ,, | 51 | 34 | 31 | 15 | 8 | 2 | — | 0 |
| ,, | Buffalo | 36 | 7 | 4 | 2 | 1 | 0 | — | 1 |
| ,, | $C_{57}$ | 50 | 46 | 39 | 38 | 23 | 15 | — | 4 |
| 6-hydroxy-$\Delta^4$-cholestene-3-one (in olive oil) | stock | 50 | 35 | 26 | 13 | 9 | 0 | — | 1 |
| (in sesame oil) | ,, | 54 | 30 | 23 | 21 | 4 | 0 | — | 1 |
| $\Delta^5$-cholestene-3-one (in olive oil) | ,, | 40 | 14 | 12 | 8 | 4 | 0 | — | 0 |
| ,, | $C_{57}$ | 50 | 38 | 34 | 29 | 23 | 11 | — | 1 |
| Lathosterol | $C_{57}$ | 50 | 39 | — | 17 | — | 5 | — | 0 |

TABLE IV.—*Sarcoma Induction by Purified Cholesterol (Schwenk Process) Injection in $C_{57}$ Mice Compared with Negative Results in Stock Mice.*

| No. of mice | Strain | Survivors (months) 12 | 18 | 24 | 30 | Sarcomas |
|---|---|---|---|---|---|---|
| 57 | Stock | 25 | 14 | 5 | 1 | 0 |
| 50 | $C_{57}$ | 47 | 31 | 24 | 7 | 3 (latent periods 12, 18, 24 months) |

As far as the results go they suggest that the hydroperoxide and pure cholesterol have about the same order of carcinogenic activity (Tables III and IV) which gives no support to the idea expressed by Fieser *et al.* (1955), and is implicit in the writings of Bischoff *et al.* (1955), that cholesterol is a pre-carcinogen when injected into the mouse and has to be converted to an oxidised carcinogenic derivative before it can induce sarcoma.

(III) *Trace contamination by carcinogens of hydrocarbon type*

The experiments designed to test lipoid tissue-extracts and the earlier tests with cholesterol before 1950 were carried out in a room at the Chester Beatty Institute where some other series of mice were being treated with potent carcinogens such as benzpyrene, thus introducing the possibility of contamination. In 1950 all the experiments on carcinogenesis by lipoids and steroids were transferred to our country laboratories, twenty miles out of London, where the atmosphere is less smoky and where there are arrangements for housing the mice in rooms uncontaminated by carcinogens other than cholesterol (Hieger and Orr, 1954). Nevertheless, it soon became clear that even if all reasonable precautions were taken to avoid contamination, cholesterol still induced sarcoma in mice. In order to obtain some data on the degree of contamination which might appreciably affect the yield of sarcomas with cholesterol alone, series of mice were given small doses of benzpyrene to find where the threshold dose of benzpyrene lay (Table V).

The results of the experiments detailed in Table V suggest that (1) the threshold dose of benzpyrene is of the order of 1 $\gamma$ for $C_{57}$ mice or stock mice, (2) an amount of benzpyrene in the region of 10 $\gamma$ would have to be injected along with the cholesterol in order to give a yield of sarcomas appreciably higher than what would be expected with cholesterol alone, (3) the sensitivity of mice to small doses of benzpyrene can vary by a factor of at least 5 to 1.

TABLE V.—*Threshold Dose of Benzpyrene Required for Sarcoma Induction*

| Experiment | No. of mice at start | Strain | Total dose of benzpyrene (in 10% cholesterol : 90% olive oil) | How administered | No. of sarcomas |
|---|---|---|---|---|---|
| 1 | 50 | $C_{57}$ | 0·04 $\gamma$ | in a single dose | 0 |
| 2 | ,, | ,, | 0·4 $\gamma$ | ,, | 1 |
| 3 | ,, | ,, | 4·0 $\gamma$ | ,, | 5 |
| 4 | ,, | ,, | 40·0 $\gamma$ | ,, | 23 |
| 5 | ,, | stock | 7·5 $\gamma$ | divided in 6 injections | 2 |
| 6 | ,, | ,, | 0·75 $\gamma$ | ,, | 3 |
| 7 | ,, | ,, | 0·075 $\gamma$ | ,, | 3 |
| 8 | ,, | ,, | 50 $\gamma$ | ,, | 19 |
| 9 | ,, | ,, | 5 $\gamma$ | ,, | 3 |
| 10 | ,, | ,, | 0·5 $\gamma$ | ,, | 0 |
| 11 | ,, | ,, | 0 | ,, | 0 |
| 12 | ,, | ,, | 0 | ,, | 0 |
| 13 | ,, | ,, | 10 $\gamma$ | ,, | 1 |
| 14 | ,, | $C_{57}$ | 10 $\gamma$ | ,, | 10 |
| 15 | ,, | stock | 10 $\gamma$ | in single dose | 6 |

If it be assumed as a working hypothesis that sensitivity is proportional to the yield of tumours and inversely as the dose of carcinogen required, experiments 3, 13, 14 and 15 (Table V) suggest that $C_{57}$ mice are of the order of 10 times as sensitive as stock mice to threshold doses of benzpyrene when it is administered in 5 divided doses, but only about twice as sensitive when the dose is given in one injection ; experiments 9 and 13 suggest that the sensitivity of different lots of stock mice can vary by something of the order of 6 : 1 or more. Although the benzpyrene dose varied in the ratio 1 : 100 in experiments 5, 6, 7, the yield of tumours was much the same, suggesting that the cholesterol in the injection material was the active carcinogen and not the benzpyrene. In these three experiments the incidence was about 6 per cent calculated on the number of mice at start, but about double this figure when the calculation is based on the mice which survived a minimum of 1 year and therefore were effective, (i.e. at risk). An incidence of 10–15 per cent would agree well with that obtaining in several of our experiments where the mice developed a reasonable proportion of tumours when injected with cholesterol (the frequently occurring incidence of about 10–15 per cent will be further discussed in a forthcoming publication). This figure of 10–15 per cent can now be contrasted with the results for experiments 10, 11, 12 where the incidence was 0 per cent. As far as the data go, they suggest that the sensitivity of different batches of mice can vary over a considerable range whether this sensitivity is assayed by the use of cholesterol or by small doses of benzpyrene.

To extend the idea of possible contamination by benzpyrene a further step, the following experiment was carried out : mice were injected with cholesterol and arranged in groups of 4 in separate cages ; to each cage was then added a single mouse of a different colour (a coloured mouse with 4 white mice or a white mouse with 4 coloured mice) which was painted biweekly with benzpyrene solution in the interscapular area during the whole course of the experiment. The mice injected with cholesterol were thus in an environment contaminated with benzpyrene, yet only in experiment A (Table VI) was there a large number of sarcomas which would be attributed to benzpyrene. In this particular experiment, the 11 injections of cholesterol were given at 4 weekly intervals concurrently with the biweekly painting of the single mouse with benzyprene. A possible explanation of the results in experiment A is that at the injection of cholesterol some benzpyrene on the fur, transferred there by contact with the painted mouse, was pushed through the skin into the subcutaneous layers. In a few cases papillomas arose on the skin of the mice which had not been painted with benzpyrene but had a cage-mate which was so treated.

TABLE VI.—*Effect of Exposing Mice Injected with Cholesterol to an Environment Contaminated with Benzpyrene*

In each experiment below the mice were caged in groups of 5. Four mice were for injection *only*, and the fifth for painting *only* (with benzpyrene).

| | | Strain | Survivors: Start | 12 (months) | 18 | 24 | Sarcomas |
|---|---|---|---|---|---|---|---|
| A | Injected 11 times with 10 per cent cholesterol in olive oil at beginning of each of 11 months. In the 2nd and 3rd week of each month—biweekly painting | stock | 80 | 42 | 16 | — | 12 |
| B | 4 injections (cholesterol) at fortnightly intervals, then after a 3-week interval painting begun | $C_{57}$ | 40 | 30 | 26 | 3 | 0 |
| C | 4 injections (cholesterol) at fortnightly intervals, then after a 3-week interval—painting begun. | stock | 40 | 30 | 13 | 2 | 2 |
| D | 4 injections (cholesterol) at fortnightly intervals, then after 4 months—painting begun. | stock | 40 | 28 | 12 | 2 | 0 |
| E | Paint biweekly for 3 months only. Then after an interval of 1 month, 4 fortnightly injections of olive oil—tristearin (5 : 1). | stock | 40 | 12 | 2 | — | 0 |
| F | Inject 4 times with an olive oil : tristearin mixture (5 : 1), then after an interval of 3 weeks, biweekly painting begun. | stock | 44 | 28 | 14 | 2 | 2 |
| G | Painted biweekly for 3 months. Then, after an interval of 2 weeks given 4 injections of cholesterol. | stock | 60 | 11 | — | — | 0 |

### EXPLANATION OF PLATES

FIG. 1.—Sarcoma induced at site of injection of purified cholesterol in olive oil. ♀ stock mouse. 16th month. × 65.

FIG. 2.—Same tumour as in Fig. 1. × 300.

FIG. 3.—Sarcoma induced at site of injection of purified cholesterol in olive oil. ♂ stock mouse. 18th month. × 65.

FIG. 4.—Same tumour as Fig. 3. × 300.

FIG. 5.—Sarcoma induced at site of injection of purified cholesterol in olive oil. ♂ stock mouse. 9th month. × 75.

FIG. 6.—Same tumour as in Fig. 5. × 300.

FIG. 7.—Tumour on *left* side of ♀ stock mouse which had been injected on *right* side with tristearin in olive oil. 19th month. × 90.

FIG. 8.—Same tumour as in Fig. 6. × 300.

FIG. 9.—Tumour on *left* side of ♀ stock mouse which had been injected on *right* side with cholesterol in olive oil containing added 1 per cent turpentine. 19th month. × 90.

FIG. 10.—Same tumour as in Fig. 9. × 300.

FIG. 11.—Tumour on *left* side of ♂ stock mouse which had been injected on *right* side with cholesterol in olive oil. 15th month. × 90.

FIG. 12.—Same tumour as in Fig. 11. × 300.

1

2

3

4

Hieger.

5

6

7

8

Hieger.

9

10

11

12

Hieger.

These data show that no very stringent precautions need to be taken to avoid fictitious results due to contamination, since the mice in experiments B and C, although in an environment contaminated with benzpyrene, did not develop sarcomas in excess of what would be expected from the injection of cholesterol *per se*. It is difficult to explain the appearance of the two sarcomas in experiment F (Table VI) where no cholesterol but a mixture of two fats were used for injection (olive oil and tristearin) in order to test the possible localising effect of lipoid substances other than cholesterol.

(IV) *In other laboratories, cholesterol has not produced tumours*

Hartwell (1951) quotes 39 entries under the section dealing with tests for the carcinogenicity of cholesterol ; some details are given of the species of experimental animal, vehicle and dose of cholesterol, site and route of administration, duration of experiment, survival rate and yield of tumours. None of these 39 experiments gave positive results of the kind which are the criteria of the present enquiry, i.e. spindle-celled sarcoma at the site of injection (sub-cutaneous) in mice injected with large doses of cholesterol of the order of a minimum of 40 mg. administered in lard or olive oil, in mice of stock or $C_{57}$ strain which had a satisfactory survival rate. However, Hartwell's compilation was not completely up to date even in 1951 since he does not refer to the writer's experiments published in 1947 where 11 sarcomas were obtained by the injection of commercial cholesterol in 144 mice (Hieger, 1947).

As far as the author is aware, his experiments are almost the only ones giving positive results in tests for the carcinogenic activity of cholesterol. Is the discrepancy due to differences in the solvent, in the strain of mouse, in the duration of the experiment or in the environmental conditions of the animals ? The role played by the solvent in carcinogenesis by lipoid substances and steriods has not yet been settled. Cholesterol of different degrees of purity has been found highly active when administered in olive oil or in lard ; Steiner (1941) reported that mixtures of the unsaponifiable fractions of human liver with tricaprylin or with sesame oil were strongly carcinogenic ; Bischoff *et al.* (1955) tested oxidative derivatives of cholesterol suspended in sesame oil with positive results. These four oils (lard, olive oil, sesame oil and tricaprylin) can accordingly be considered as suitable vehicles for testing carcinogens of lipoid or steroid type since the control tests on the oils alone give a very low yield of tumours, namely, of the order of ½ per cent (Table I).

The choice of the strain of experimental mouse is probably an important factor in affecting the response to lipoid carcinogens. Cholesterol in olive oil or in lard has given an incidence of 10–15 per cent or more of sarcomas in $C_{57}$ mice, in mixed commercial stock mice, and in the laboratory stock bred from a mixed commercial stock. In an experiment on 100 mice of the CBA strain injected with commercial cholesterol in olive oil, the yield of sarcomas was less, namely 2 per cent (Table VII). Small series of $C_3H$ mice tested with the same solution proved refractory and small series of mice of the MRC strain reacted positively.

Such differences of sensitivity of different strains of mice to cholesterol as a carcinogen are not unexpected since variations of susceptibility to conventional carcinogens (e.g. hydrocarbon type) are well known and an example is shown in Table V. What is more difficult to explain is the frequently occurring fluctuations in response exhibited by different batches of mice of the same genetic origin.

Differences of sensitivity to carcinogens can easily be found when a minimal dosage of carcinogen is used but when an excessive dose of carcinogen is applied (such as, say, a milligram or even a tenth of a milligram of benzpyrene) anything but gross differences of sensitivity will be masked by the excess of carcinogen ; therefore, in order to reveal and assess the comparative sensitivity of different groups of mice, the dose of carcinogen should be at threshold, or near threshold, level. If the carcinogen is of an order of potency much below that of the hydrocarbon carcinogens of the type of benzpyrene, it can be expected that even after the application of large doses, only a fraction of the mice would react and the conditions would be well suited for demonstrating variations of susceptibility which are not likely to appear when hydrocarbon carcinogens are employed, unless in minute doses. In Table VII are shown the fluctuations of response when mice of $C_{57}$ or stock strains were tested with pure or with commercial cholesterol. The yield of sarcomas varied from 0–14 per cent or more in different batches of mice. Experiments t and q (both groups injected with commercial cholesterol) are of special interest : the origin of the mice was the same (a more complete correspondence would have been achieved if the individual members of the two groups had been thoroughly " shuffled "). Group q was housed in a room kept at about 70° F. and water was supplied in bulbs in the usual way : group t was housed in cages well supplied with wood wool, in a room facing north where the temperature followed the external temperature but was not allowed to go below 40° F. and the only water available to the mice was supplied in cabbage leaf. In experiment q the yield of sarcomas was zero in 50 mice, in t it was 5 in 46 mice (initially) amounting to an incidence of 16 per cent calculated on the survivors at one year.

These experiments suggest that environmental factors were of importance in influencing the response to the carcinogen. A comparison of experiments s (pure cholesterol) and q (commercial cholesterol) suggests that purified cholesterol (Schwenk process, see Hieger (1957) for details) is more potent carcinogenically than commercial cholesterol ; but it must be noted that in experiment r no sarcomas were induced in 54 stock mice with the olive oil preparation of pure cholesterol, whereas in n and o the incidences were 14 per cent in one case and was calculated to have been the same theoretically in the other (Hieger, 1957). It is very difficult to find any explanation of why the same, or very nearly the same, preparation of cholesterol gives 14 per cent of sarcomas in stock mice in one experiment and 0 per cent in another.

The conclusions to be drawn from the data shown in Table VII are that no single variable—nor the purity of the cholesterol, nor chance contamination with atmospheric carcinogens nor strain differences, nor solvent differences—can be implicated as solely responsible for the fluctuations in carcinogen response ; the alternative explanation is that a combination of some genetic, congenital or environmental factors (e.g. temperature and drinking water) are the effective agents, but of course such an explanation has still to be proved adequate.

In Section III the range of variations in susceptibility to threshold doses of benzpyrene was observed to be something of the order of 5 : 1, if the sensitiveness be regarded provisionally as proportional to the percentage yield of tumours and inversely as the dose of carcinogen. Fluctuations of susceptibility of this order of magnitude could explain why some batches of mice do not react under the stimulus of cholesterol as carcinogen ; as a first approximation it is suggested that such variations are due to the operation of the same factors which cause variations in the incidence of " spontaneous " neoplasia in mice, although of course, there is no experimental evidence as yet for this idea. It will be recalled that Tannenbaum and Silverstone (1957) showed that mere alterations in the calorific value of the diet of mice could greatly influence the incidence of spontaneous mammary cancer and of tumours induced with carcinogens.

TABLE VII.—*Table Showing Incidence of Sarcomas in Mice After Subcutaneous Injection with Cholesterol*

| Experiment | Institute | Room | Strain | Pure or commercial (comm.) Cholesterol | No. of mice and survivors (months) Initial | 12 | 18 | 24 | Sarcomas | % Incidence on initial No. | Calculated on survivors at 1 year % |
|---|---|---|---|---|---|---|---|---|---|---|---|
| a | C.B. | — | $C_{57}$ | comm. | 108 | 68 | 45 | 10 | 10 | 9·3 | 14 |
| b | ,, | — | stock | ,, | 45 | 18 | 5 | 3 | 5 | 11 | 28 |
| c | ,, | — | MRC | ,, | 20 | 16 | 8 | 2 | 2 | 10 | 12 |
| d | ,, | — | $C_{57}$ + stock | pure | 34 | 18 | 11 | 2 | 1 | 3 | 6 |
| e | P.W. | non-C | $C_{57}$ | comm. | 60 | 28 | 14 | 3 | 1 | 1·7 | 3·6 |
| f | ,, | ,, | stock | ,, | 70 | 38 | 18 | 2 | 3 | 4·3 | 7·9 |
| g | ,, | ,, | ,, | ,, | 65 | 57 | 41 | 11 | 3 | 4·6 | 5·4 |
| h | ,, | C | ,, | ,, | 100 | 75 | 45 | 10 | 8 | 8 | 10·7 |
| i | ,, | ,, | CBA | ,, | 100 | 71 | 45 | 2 | 2 | 2 | 2·8 |
| j | ,, | non-C | $C_{57}$ | pure | 172 | 126 | 81 | 20 | 5 | 2·9 | 4 |
| k | ,, | ,, | ,, | comm. | 30 | 10 | 7 | 5 | 1 | 3·3 | 10 |
| l | ,, | ,, | ,, | ,, | 39 | 24 | 16 | 11 | 3 | 7·7 | 13 |
| m | ,, | C | ,, | ,, | 75 | 46 | 26 | 3 | 4 | 5·5 | 8·7 |
| n | ,, | ,, | stock | pure | 115 | 66 | 22 | 10 | 11 | 9·5 | 14 |
| o | ,, | non-C | ,, | ,, | 109 | all † < 1 yr. (epidemic) | | | 3 | 3 | probably much > 3 |
| p | ,, | ,, | ,, | comm. | 53 | 27 | 11 | 0 | 0 | 0 | — |
| q | ,, | ,, | $C_{57}$ | ,, | 50 | 38 | 32 | 23 | 0 | 0 | — |
| r | ,, | ,, | stock | pure | 53 | 25 | 12 | 2 | 0 | 0 | — |
| s | ,, | ,, | $C_{57}$ | ,, | 50 | 47 | 35 | 22 | 3 | 6 | 6·4 |
| t | ,, | low temperature | $C_{57}$ | comm. | 46 | 32 | 23 | 12 | 5 | 11 | 16 |
| u | ,, | ,, | stock | ,, | 40 | 19 | 5 | 0 | 0 | — | — |

C.B. = Chester Beatty Research Institute, London. P.W. = Pollards Wood in Buckinghamshire, 20 miles out of London. Non-C = Non-Carcinogen Room. Carcinogens of conventional type (e.g. benzpyrene) were neither used nor kept in this room. C = Carcinogen room. In this room, besides mice injected with cholesterol, were other series undergoing treatment with benzpyrene. Low-temperature room = This room faces north; temperature not below 40°F. but otherwise follows outside temperature. Water is supplied only as present in fresh cabbage leaf.

Dunn, Heston and Deringer (1956) reported that in the female mice in their colony consisting of $C_3H$ strain bearing the mammary milk agent, $C_3Hf$ lacking the agent, $C_{57}BL$ strain and $F_1$ and backcross hybrids of these strains, a total yield of 106 subcutaneous fibrosarcomas were found in 4049 mice (initially). These workers quote Cloudman (1941) who, referring to a certain colony of mice stated that " sarcomas were among the more common malignant tumours in the subcutaneous regions, but there were no high-tumour stock, and it was unusual to find an incidence of over 15 per cent." Dunn, Heston and Deringer's (1956) results (106 tumours in 4049 female mice) represent a crude incidence of $2\frac{1}{2}$ per cent sarcomas, but as the average latent period when the tumours occurred was at the advanced age of 22–32 months, which was approximately the average longevity of the mice, the true incidence was very probably a multiple of $2\frac{1}{2}$ per cent, say 5 per cent or more, since a fair proportion were dead before reaching the latent period and could not be considered at risk.

Some impression of the variations of incidence in their experiments is conveyed by the number of sarcomas in consecutive groups of $C_3$Hf mice (Table VIII).

TABLE VIII.—*Spontaneous Fibrosarcoma in $C_3$Hf Mice* (*Dunn, Heston and Deringer*, 1956)

| Genetic composition of foster mother | Number of mice | Number of sarcomas |
|---|---|---|
| (a) $C_3$Hf mice foster-nursed on ⟶ $C_{57}$ (100 per cent $C_{57}$ chromatin : 0 per cent $C_3$H chromatin) | 81 | 3 |
| (b) $C_3$Hf mice foster-nursed on ⟶ Cross-bred (50 per cent $C_{57}$ chromatin : 50 per cent $C_3$H chromatin) | 90 | 9 |
| (c) $C_3$Hf mice foster-nursed on ⟶ Cross-bred (25 per cent $C_{57}$ chromatin : 75 per cent $C_3$H chromatin) | 64 | 0 |

It is very difficult to explain how the variations in the genetic composition of the foster-mothers of the mice in experiments b and c was sufficient to change the incidence of spontaneous sarcoma from a maximum of 10 to 0 per cent.

The foregoing considerations suggest that (1) at low intensity stimulus (cholesterol as carcinogen, or threshold doses of benzpyrene or " spontaneous " sarcoma) several factors together or separately can cause wide fluctuations of incidence of sarcoma ; (2) that of these factors, two can be identified namely the " environment " and the genetic composition of the mice ; (3) that alternatively some other factors are operative, the nature of which is as yet unknown.

These 3 possibilities could explain the disagreement between the results of tests on cholesterol and its oxidative derivatives which were used by Bischoff, *et. al.* (1955) and by the writer. Bischoff *et. al.* (1955) and Fieser *et al.* (1955) report that $6\beta$-hydroperoxy-$\Delta^4$-cholestene-3-one is a potent carcinogen, for it gives sarcoma in over 60 per cent of Buffalo mice (Table II). In the tests described in Table III this compound is shown to be as active as cholesterol, i.e. giving a sarcoma incidence of 8–10 per cent but these experiments were carried out on $C_{57}$ mice. A batch of Buffalo mice imported from the U.S. appeared healthy on arrival but have not maintained this condition, they are very prone to mite infestation (however, vigorous treatment with DDT and with benzyl-benzoate seems to be effecting some improvement of the state of the skin). Bischoff and Rupp (1946) find that this strain of mice is liable to spontaneous sarcoma, they state " . . . In previous experiments male mice observed for 20 months or more developed a higher incidence of skin tumours ; namely 10 per cent in 30 control mice, and 23 per cent in 30 males that had received oestrone." Perhaps the Buffalo mice are specially sensitive to some carcinogens since their subcutaneous tissues are prepared, as it were, for neoplasia, assuming that the two processes follow the same path, but of course, their insensitivity to cholesterol as carcinogen would remain unexplained.

The spontaneous subcutaneous sarcoma rate in the mice in the author's laboratory must be very small indeed. An estimate of the incidence could be made by a count of the sarcomas which have appeared on the *left* side of the animals. All the injections of cholesterol, derivatives of cholesterol, lipoid fractions of tissues and controls on solvents, were carried out on the *right* side. To date over 140 sarcomas have been recorded on the right side and 7 subcutaneous tumours on the left side, but the histological appearance of most of these 7 tumours is rather different from that of the spindle-celled sarcomas induced at the site of injection by cholesterol and by the lipoid tissue-extracts which are very similar histologically to the sarcomas produced by hydrocarbon-type carcinogens. Consequently, it is doubtful if there is any appreciable incidence of spontaneous spindle-celled sarcoma in our stocks. Photomicrographs of these two kinds of subcutaneous tumour are shown in Fig. 1–12.

Guérin (1954) states that in his colony of 6000 mice the most frequently occurring spontaneous tumour was not mammary cancer but connective tissue tumours of the reticuloendothelial system (275 cases equivalent to an incidence of 4 per cent). Thirteen spontaneous sarcomas were found, 12 of which were " fusohistiocytaire " ; the average tumour age was about 20 months.

V. *Role of heating as a possible contributory factor in carcinogenic activity of solution of cholesterol.*

Cholesterol as large dense flakes dissolves slowly on heating with olive oil at water bath temperature and when a large series of mice (say 50–100) are to be injected in turn, the time required for total heating may be approximately 2 hours. During that time chemical changes may occur in the solution ; indeed the solutions and even oils alone (particularly sesame oil and lard) after being heated on the water bath show a much increased spectroscopic absorption in the ultraviolet region of the spectrum.

Since our experiments with cholesterol administered in aqueous medium (i.e. finely ground cholesterol in suspension in 4 per cent gelatin gel) have so far given negative results, the criticism has been put forward that chemical changes taking place or initiated during the heating of the cholesterol with oil could explain the carcinogenic activity of the solution and the inactivity of the non-oily suspension. However, there is a total lack of experimental evidence for (and some facts can be advanced which do not support) this possibility. The control tests on the oily solvents alone (olive oil and lard) comprised over 1,000 mice which produced 5 sarcomas only. The oils were heated on the water bath before injection but since they were either liquid or melted very quickly, the time of heating was mainly dependent on the number of mice being injected and therefore the total heating could have been less than in the experiments with solutions of cholesterol ; but since the total number of injections was greater in the control series because oil alone is more readily dispersed in the tissues, the effective total heating was probably not very different in the two cases.

To pursue the same line of argument further, it would now be necessary to postulate that the presence of cholesterol during the heating is necessary for inducing the hypothetical chemical change. Sarcomas were not obtained in experiments set up to test this possibility (Table IX). Positive results must take precedence over negative results ; the latter may be due to an insensitive batch of mice or to environmental conditions which are not conducive to sarcoma production : some solutions of cholesterol heated in the usual way have *not* produced sarcoma in mice, and if for the sake of argument it be assumed that the agent is indeed formed during the heating, its hypothetical presence therefore is not as decisive as e.g. the sensitivity of the mice or the environmental factor. There seems no reason for multiplying the number of unknowns, and the formation of a carcinogen by heating oil and cholesterol at water bath temperature remains at present unproved, what evidence exists is against such possibility.

TABLE IX.—*Results of Injecting* (1) *Heated Olive Oil*; (2) *Heated Solution of Cholesterol* (10 *per cent*) *in Olive Oil*

| Number of Preparation | No. of mice | Strain | | Survivors (months) 12 | 18 | 24 | Sarcomas |
|---|---|---|---|---|---|---|---|
| (1) | 12 | $C_{57}$ | 50 | 35 | 17 | 6 | 0 |
| | 38 | stock | | | | | |
| (2) | 17 | $C_{57}$ | 50 | 35 | 15 | 4 | 0 |
| | 33 | stock | | | | | |
| (3) | 14 | $C_{57}$ | 50 | 38 | 16 | 9 | 1 |
| | 36 | stock | | | | | |

(1) Olive oil heated, with access of air, 75 hours on boiling water bath.
(2) 10 per cent cholesterol + 90 per cent olive oil heated with access of air, 75 hours on boiling water bath.
(3) Olive oil heated, with access of air, 76 hours on boiling water bath then 10 per cent cholesterol added without prolonged further heating.

### SUMMARY AND CONCLUSIONS

1. Over 140 sarcomas in mice have been induced by the subcutaneous injection of oily solutions of (1) the unsaponifiable fraction of human tissue, or (2) of cholesterol.

2. Purified cholesterol is at least as potent as the commercial product before purification.

3. In one experiment on highly purified cholesterol, 11 sarcomas developed in 115 mice of which 66 lived over 1 year. If the mice which died early and were not at risk be omitted the incidence amounts to 14 per cent.

4. One of the oxidative derivatives of cholesterol reported by Fieser and Bischoff to be carcinogenic has given positive results when tested in the writer's laboratory. The most active of their compounds, 6$\beta$-hydroperoxy-$\Delta^4$-cholestene-3-one, which is reported by Bischoff to give 60 per cent sarcomas in Buffalo mice, has induced sarcoma in 4 out of 50 treated $C_{57}$ mice.

5. The degree of response to cholesterol as carcinogen has been found to fluctuate widely. It is suggested that such variations of sensitiveness are revealed by carcinogenic stimuli of low-intensity, and are linked with congenital and environmental factors.

This investigation has been supported by grants to the Chester Beatty Research Institute from the British Empire Cancer Campaign, the Jane Coffin Childs Memorial Fund for Medical Research, the Anna Fuller Fund, and the National Cancer Institute of the National Institutes of Health, United States Public Health Service.

### REFERENCES

BISCHOFF, F., LOPEZ, G. AND RUPP, J. J.—(1954) *Abstr. Amer. chem. Soc.* March 3–C.
*Idem*, LOPEZ, G., RUPP, J. J. AND GRAY, C. L.—(1955) *Fed. Proc.*, **14**, 183.
*Idem* AND RUPP, J. J.—(1946) *Cancer Res.*, **6**, 403.

FIESER, L. F., GREENE, T. W., BISCHOFF, F., LOPEZ, G. AND RUPP, J. J.—(1955) *J. Amer. chem. Soc.*, **77**, 3928.
CLOUDMAN, A. M.—(1941) Spontaneous neoplasms in mice. *In* ' Biology of the Laboratory Mouse ' (Snell, G. D. ed.). Philadelphia (Blakiston).
DUNN, T. B., HESTON, W. E. AND DERINGER, M. K.—(1956) *J. nat. Cancer Inst.*, **17**, 639.
GUÉRIN, M.—(1954) ' Tumours Spontanées des Animaux de Laboratoire '. Paris (Legrand).
HARTWELL, J. L.—(1951) ' Survey of compounds which have been tested for carcinogenic activity '. Bethesda, Md. (National Cancer Institute).
HIEGER, I.—(1940) *Amer. J. Cancer*, **39**, 496.—(1947) *Nature*, **160**, 270.—(1949) *Brit. J. Cancer*, **3**, 123.—(1957) *Proc. Roy. Soc. B*, **147**, 84.—(1959) *Acta Un. int. Cancr.* (in press).
*Idem* AND ORR, S. F. D.—(1954) *Brit. J. Cancer*, **8**, 274.
DES LIGNERIS, M. J. A.—(1940) *Amer. J. Cancer*, **39**, 489.
SANNNIÉ, C., TRUHAUT, R., GUÉRIN, M. AND GUÉRIN, P.—(1941) *Bull. Ass. franç. Cancer*, **29**, 106.
SHABAD, L.—(1937) *C.R. Soc. Biol., Paris*, **124**, 213.
STEINER, P. E.—(1941) *Cancer Res.*, **1**, 750.
TANNENBAUM, A. AND SILVERSTONE, H.—(1957) ' Cancer ' I (ed. R. W. Raven) London (Butterworths) p. 306.